# Supplementary material for: Causal Relationship between Aging and Anorexia Nervosa: A White-Matter-Microstructure-Mediated Mendelian Randomization Analysis
Source: Biomedicines. 2024 Aug 16;12(8):1874. doi: 10.3390/biomedicines12081874 (PMC11351342; doi:10.3390/biomedicines12081874)
Supplement: Supplementary file 1 [file biomedicines-12-01874-s001.zip › Supplementary Figure/Figure S4/Singe SNP plot of MR analysis for TL to ubm-b-2041.pdf]

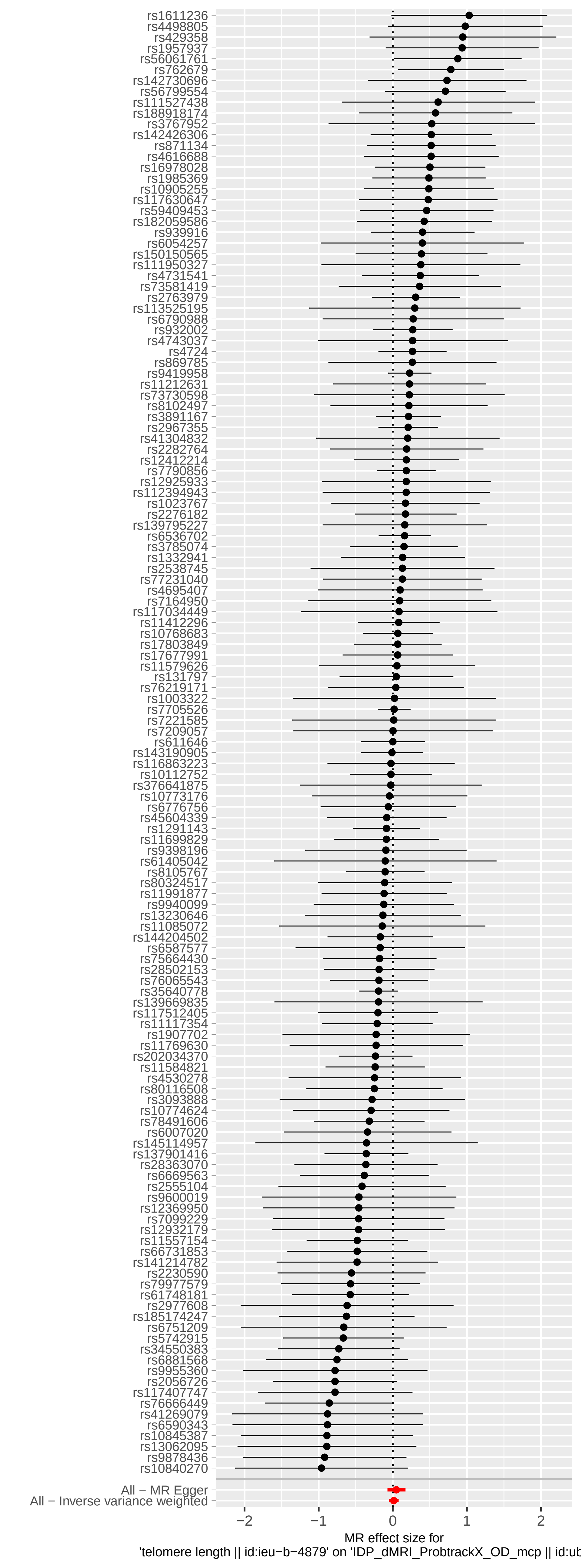
rs1611236
rs4498805
rs429358
rs1957937
rs56061761
rs762679
rs142730696
rs56799554
rs111527438
rs188918174
rs3767952
rs142426306
rs871134
rs4616688
rs16978028
rs1985369
rs10905255
rs117630647
rs59409453
rs182059586
rs939916
rs6054257
rs150150565
rs111950327
rs4731541
rs73581419
rs2763979
rs113525195
rs6790988
rs932002
rs4743037
rs4724
rs869785
rs9419958
rs11212631
rs73730598
rs8102497
rs3891167
rs2967355
rs41304832
rs2282764
rs12412214
rs7790856
rs12925933
rs112394943
rs1023767
rs2276182
rs139795227
rs6536702
rs3785074
rs1332941
rs2538745
rs77231040
rs4695407
rs7164950
rs117034449
rs11412296
rs10768683
rs17803849
rs17677991
rs11579626
rs131797
rs76219171
rs1003322
rs7705526
rs7221585
rs7209057
rs611646
rs143190905
rs116863223
rs10112752
rs376641875
rs10773176
rs6776756
rs45604339
rs1291143
rs11699829
rs9398196
rs61405042
rs8105767
rs80324517
rs11991877
rs9940099
rs13230646
rs11085072
rs144204502
rs6587577
rs75664430
rs28502153
rs76065543
rs35640778
rs139669835
rs117512405
rs11117354
rs1907702
rs11769630
rs202034370
rs11584821
rs4530278
rs80116508
rs3093888
rs10774624
rs78491606
rs6007020
rs145114957
rs137901416
rs28363070
rs6669563
rs2555104
rs9600019
rs12369950
rs7099229
rs12932179
rs11557154
rs66731853
rs141214782
rs2230590
rs79977579
rs61748181
rs2977608
rs185174247
rs6751209
rs5742915
rs34550383
rs6881568
rs9955360
rs2056726
rs117407747
rs76666449
rs41269079
rs6590343
rs10845387
rs13062095
rs9878436
rs10840270
All − MR Egger
All − Inverse variance weighted
−2
−1
0
1
2
MR effect size for
'telomere length || id:ieu−b−4879' on 'IDP_dMRI_ProbtrackX_OD_mcp || id:ub
